# *Parageobacillus thermoglucosidasius* Strain Engineering Using a Theophylline Responsive RiboCas for Controlled Gene Expression

**DOI:** 10.1021/acssynbio.3c00735

**Published:** 2024-03-22

**Authors:** Matthew S. H. Lau, Abubakar Madika, Ying Zhang, Nigel P. Minton

**Affiliations:** † BBSRC/EPSRC Synthetic Biology Research Centre (SBRC), Biodiscovery Institute, School of Life Sciences, University of Nottingham, University Park, Nottingham NG7 2RD, U.K.; ‡ Department of Microbiology, Faculty of Life Sciences, Ahmadu Bello University, Zaria 810107, Nigeria; § NIHR Nottingham Biomedical Research Centre, Nottingham University Hospitals NHS Trust and The University of Nottingham, Nottingham NG7 2RD, U.K.

**Keywords:** riboswitch, inducible systems, CRISPR/Cas9, genetic tools, thermophile, synthetic biology

## Abstract

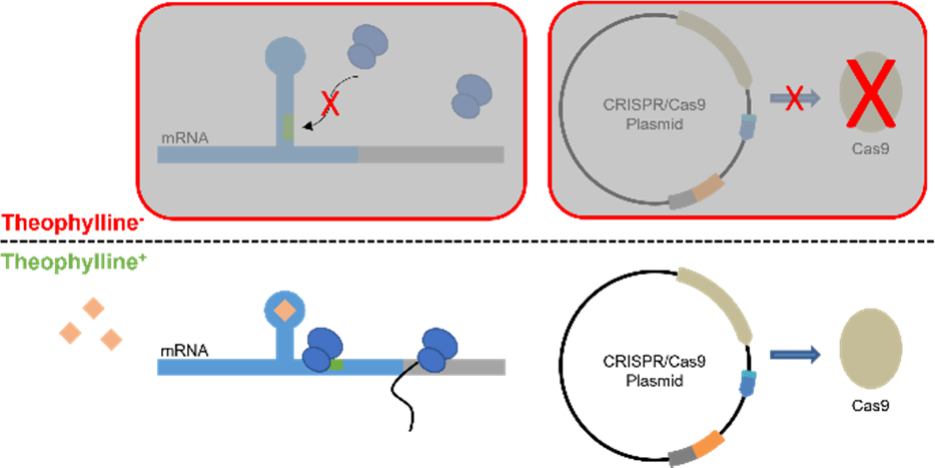

The relentless increase in atmospheric greenhouse gas concentrations as a consequence of the exploitation of fossil resources compels the adoption of sustainable routes to chemical and fuel manufacture based on biological fermentation processes. The use of thermophilic chassis in such processes is an attractive proposition; however, their effective exploitation will require improved genome editing tools. In the case of the industrially relevant chassis *Parageobacillus thermoglucosidasius*, CRISPR/Cas9-based gene editing has been demonstrated. The constitutive promoter used, however, accentuates the deleterious nature of Cas9, causing decreased transformation and low editing efficiencies, together with an increased likelihood of off-target effects or alternative mutations. Here, we rectify this issue by controlling the expression of Cas9 through the use of a synthetic riboswitch that is dependent on the nonmetabolized, nontoxic, and cheap inducer, theophylline. We demonstrate that the riboswitches are dose-dependent, allowing for controlled expression of the target gene. Through their use, we were then able to address the deleterious nature of Cas9 and produce an inducible system, RiboCas93. The benefits of RiboCas93 were demonstrated by increased transformation efficiency of the editing vectors, improved efficiency in mutant generation (100%), and a reduction of Cas9 toxicity, as indicated by a reduction in the number of single nucleotide polymorphisms (SNPs) observed. This new system provides a quick and efficient way to produce mutants in *P. thermoglucosidasius*.

The current climate crisis is driving innovation and research into alternative ways to maintain our current socioeconomic lifestyle in an environmentally conscious way. Chemicals and fuels on which the world currently depends are predominately derived from fossil fuels, and their exploitation is the principal cause of the climate change we are witnessing today. One such solution is through the utilization of microbial-based fermentations in which a renewable feedstock, such as lignocellulosic plant material, is converted into the desired products.

This has led to a period of increased attention for thermophiles as potential chassis for the production of industrially relevant chemicals and fuels through the exploitation and manipulation of the fermentative process. Thermophilic chassis offers a number of attractions over current model organisms, such as *Escherichia coli* and *Saccharomyces cerevisiae*.^1−4^

Principal among them are their ability to withstand fluctuations in temperature, pH, and environmental change and their capacity to metabolize renewable, carbohydrates derived from carbon-neutral lignocellulosic biomass. Moreover, the ability to perform a fermentative process at higher temperatures also affords several advantages. These include reduced running costs, providing optimum temperatures for enzymatic degradation of lignocellulose and the elimination of antibiotic use, the need for successive cooling and end-product inhibition through continuous distillation.^3−8^

*Parageobacillus thermoglucosidasius* (formerly *Geobacillus thermoglucosidasius*)^9^ is a thermophilic, Gram-positive bacterium and is facultatively anaerobic, with a growth range demonstrated to be between 37 and 68 °C.^10^ Like many thermophiles, *P. thermoglucosidasius* is fast-growing and can ferment both mono- and oligosaccharides, including those typically derived from lignocellulosic biomass. The current widespread use of thermophiles is hindered due to a limitation of well-developed genome editing tools, a stark contrast to their mesophilic counterparts.^11−13^ However, *P. thermoglucosidasius* is one of the few thermophiles that can be successfully genetically manipulated and in recent years has developed a suite of genome editing tools to allow the generation of engineered strains.^14−16^ Proving its worth as an industrially relevant chassis, *P. thermoglucosidasius* has already demonstrated its capability to produce biofuels from lignocellulose using genetic manipulation.^17−21^

Although the previous implementation of genome editing based on the CRISPR/Cas9 (clustered regularly interspaced short palindromic repeats/CRISPR-associated proteins) system of *Streptococcus thermophilus* in *P. thermoglucosidasius* represented a step forward on previous methods, it was not without its issues.^15^ In particular, in the current CRISPR/Cas9 system, a constitutive promoter controls the production of the *S. thermophilus* Cas9 (stCas93). The deleterious nature of stCas93 can mean that the use of constitutive promoters can often result in decreased transformation efficiencies and/or low editing efficiencies. Ideally, the production of stCas93 should be under inducible control to confine its production to when needed. This would likely lead to more rapid mutant generation, reduce the likelihood of off-target effects, and decrease the probability of mutations (single nucleotide polymorphisms, SNPs or insertions/deletions, Indels) occurring within stCas93 or the sgRNA, as well as elsewhere in the genome, and alleviate the metabolic burden of stCas93 production during the early stages of growth and transformation.

Inducible promoter systems, however, are largely absent in thermophiles, with current available options making use of metabolizable, nonspecific, or expensive inducers.^22−26^ To address these limitations, we sought to make use of riboswitches. These are composed of structured domains that form within the noncoding portions of mRNA and regulate gene expression in a ligand-dependent fashion. A riboswitch comprises an aptamer domain that has the ability to sense and bind a ligand, causing a change in the mRNA structure. This binding and subsequent conformational change affects the expression platform, modulating gene expression in ways such as transcriptional termination and translational initiation.^27^ One such riboswitch, a synthetic theophylline-responsive riboswitch, has been well characterized and shown to work successfully in several different mesophilic bacterial species.^28−35^

In this work, we were able to show that the theophylline-responsive riboswitches (E, F, and G) were active in the thermophile *P. thermoglucosidasius* at elevated temperatures. We were also able to demonstrate that we could further modulate control by adding a fourth riboswitch, riboswitch K. We showed that these riboswitches bind an inducer that is not metabolized and demonstrated that induction of the riboswitches was dosage-dependent. These riboswitches allowed us to achieve the primary goal of this work to develop a novel RiboCas93 to regulate the expression of stCas93 and demonstrate the advantages of RiboCas93 through the stable recreation of the ethanol-producing strain, AM242.

## Results and Discussion

### Construction and Testing of Theophylline-Dependent Riboswitches in *Parageobacillus thermoglucosidasius*

**Figure 1:**
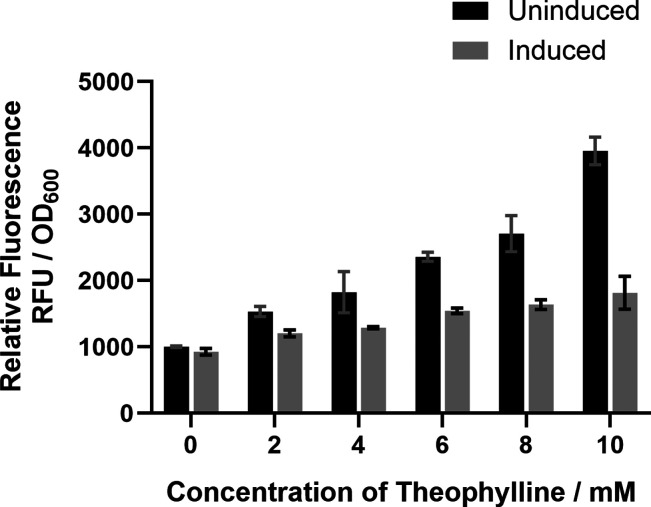
Relative strength of the constitutive promoter P_*gapdh*_ tested at different concentrations of theophylline dissolved in dimethyl sulfoxide (DMSO) (induced) and the equivalent volume of DMSO only (uninduced), in *P. thermoglucosidasius* as determined by the relative fluorescence units (RFU) of sfGFP expression, normalized by cell growth (OD_600_). Error bars denote the standard error of the mean (*n* = 3).

To achieve successful regulation of stCas93, we selected three theophylline-responsive riboswitches, riboswitch-E, -F, and -G, that had previously demonstrated a high induced expression level coupled with very low basal levels.^29^ These three riboswitches differed through the rational design of the space between the ribosomal binding sequence (RBS) and the translational start site. Therefore, we designed an additional riboswitch, riboswitch-K, using the spacer sequence from one of our, previously demonstrated, strongest RBS sequences.^15^ Using these sequences, we constructed a reporter plasmid, pMTL-RbxX (where X denotes the riboswitch) from the vector pMTL61110^20^ in which the appropriate riboswitch replaced the RBS. Upstream of the riboswitch was placed the strong promoter, P_*gapdh*_, and downstream the reporter gene, superfolder green fluorescent protein (sfGFP). The plasmid constructs were transformed into *P. thermoglucosidasius* and grown to the midexponential phase before the addition of theophylline (2 mM). After 8 h of growth, we were then able to test the level of sfGFP in the samples to determine whether the riboswitches were functional in *P. thermoglucosidasius*. However, our initial observations suggested that the solvent in which theophylline is routinely dissolved, dimethyl sulfoxide (DMSO), does not constitute an optimum solvent for testing sfGFP fluorescence-based assays. This was due to the unexpected observation that fluorescence under the control of a constitutive promoter increases with an increasing concentration of DMSO (Figure 1).

DMSO ((CH_3_)_2_SO) is an organosulfur compound polar aprotic solvent that dissolves both polar and nonpolar compounds. It is regularly used in PCR to disrupt secondary structure formation in the DNA template through local DNA denaturation by hydrogen bonding to the DNA, greatly improving the yield and specificity of PCR priming reactions.^36−38^ Indeed, enhanced RNA synthesis has also been demonstrated in the presence of DMSO, thought to be elicited by augmented initiation and elongation due to the relaxed state of the DNA generated by DMSO.^39,40^ Furthermore, the secondary structure of RNA can also be affected by DMSO which has been shown to denature RNA by reducing the *T*_m_ of the RNA^41^ and even goes as far as to reduce loops within RNA and can have a significant effect on the RNA structure and ligand binding.^42^

**Figure 2:**
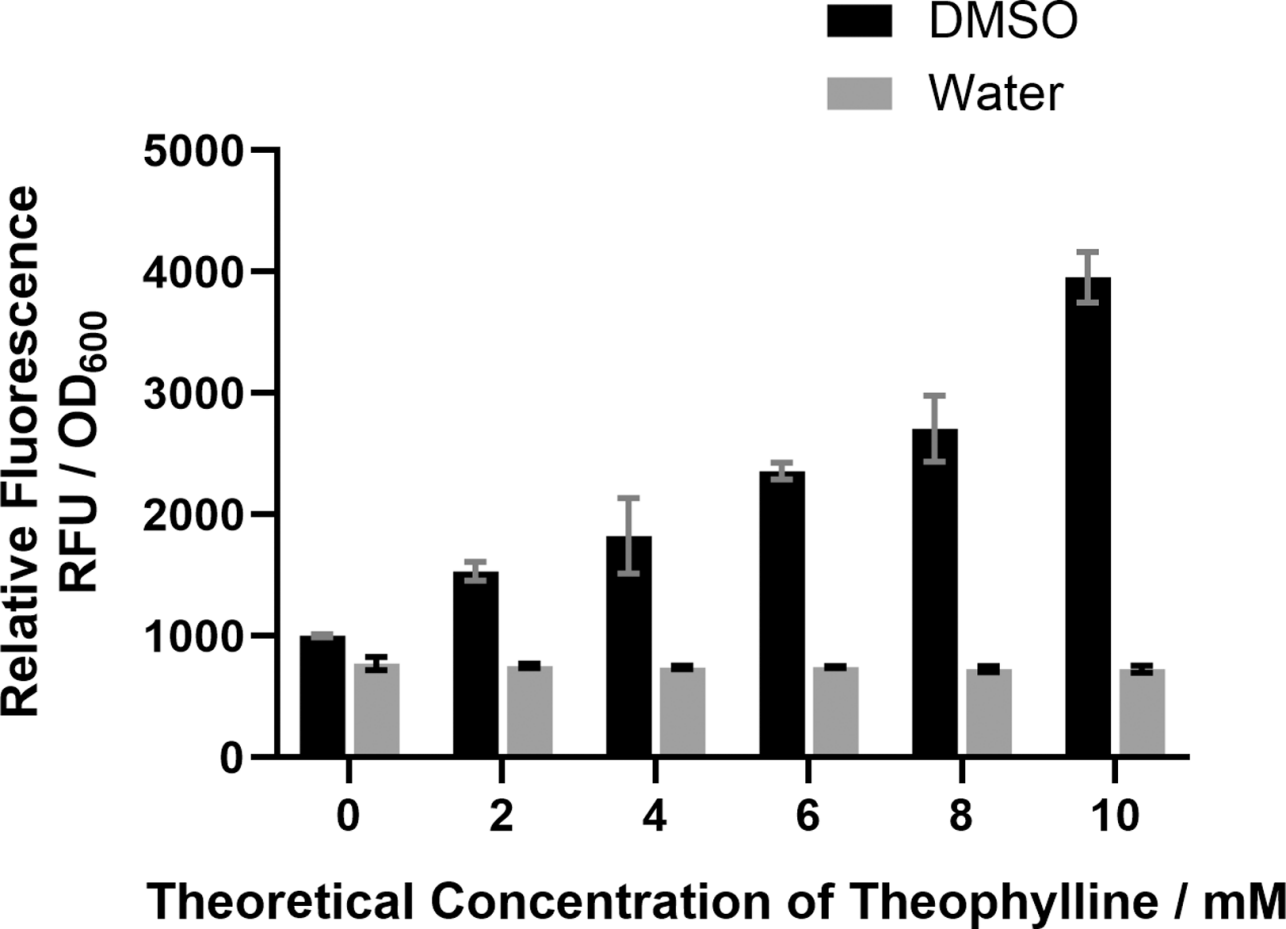
Relative strength of the constitutive promoter P_*gapdh*_ tested after grow in the equivalent volumes of DMSO and water that corresponds to the concentrations of theophylline indicated, in *P. thermoglucosidasius* as determined by the relative fluorescence units (RFU) of sfGFP expression, normalized by cell growth (OD_600_). The stock concentrations of theophylline are as follows: DMSO (48 mg/mL) and water (25 mg/mL). The volume that was added corresponded to the final concentration of theophylline in *P. thermoglucosidasius* cultures (50 mL). No theophylline was added to the cultures in this experiment. Error bars denote the standard error of the mean (*n* = 3).

Thus, we surmise that DMSO is promoting relaxation of either the DNA and/or RNA, leading to increased expression of sfGFP. Therefore, the use of DMSO in the protocol being used to test riboswitches affects the data generated by influencing the changes in the riboswitch secondary structure necessary for inhibiting expression. Furthermore, DMSO may also disrupt the aptamer-ligand binding required to induce gene expression. Consequently, we advise against the future use of DMSO when testing the relative strengths of riboswitches, despite previous work regarding these riboswitches using DMSO in their methodology. As a consequence, we redesigned the assay to use water as an alternative solvent for theophylline, after demonstrating no effect on fluorescence (Figure 2).

**Figure 3:**
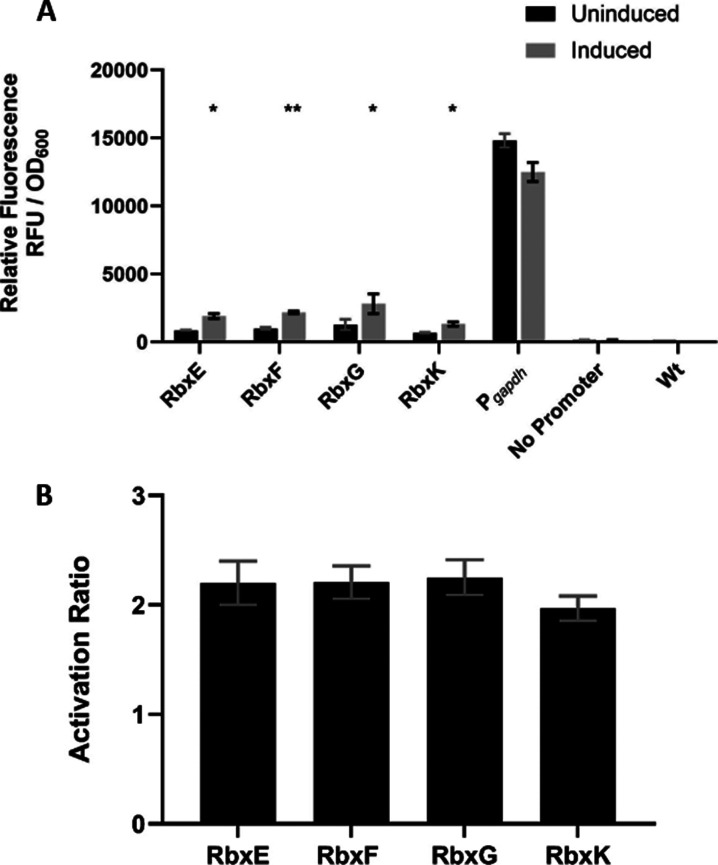
Theophylline riboswitch performance in *P. thermoglucosidasius*. (A) Relative strength of pMTL-RbxE, -F, -G, and -K, tested using 2 mM theophylline dissolved in water (induced) and the equivalent volume of water only (uninduced), in *P. thermoglucosidasius* as determined by the relative fluorescence units (RFU) of sfGFP expression, normalized by cell growth (OD_600_). P_*gapdh*_ (constitutive promoter), a promoterless sfGFP construct (no promoter), and a WT strain were used as controls. Error bars denote the standard error of the mean (*n* = 3). Statistical analysis was carried out using GraphPad Prism 9.5.1. *P* values ≤0.05 were considered statistically significant and are represented by asterisks. The following asterisk format is used throughout; *p ≤ 0.05, **p ≤ 0.01, ***p ≤ 0.001, ****p ≤ 0.0001 (unpaired two-tailed *t* test). (B) Activation ratio of the riboswitches -E, -F, -G, and -K. The activation ratio for each riboswitch was calculated by dividing the normalized RFU of sfGFP of the induced sample by the normalized RFU of the uninduced sample. Error bars denote the standard error of the mean (*n* = 3).

Subsequently, we were able to show that the riboswitches did indeed repress expression in a dose-dependent manner following the addition of theophylline (Figure 3).

### Characterization of the Theophylline-Dependent Riboswitches in *P. thermoglucosidasius*

**Figure 4:**
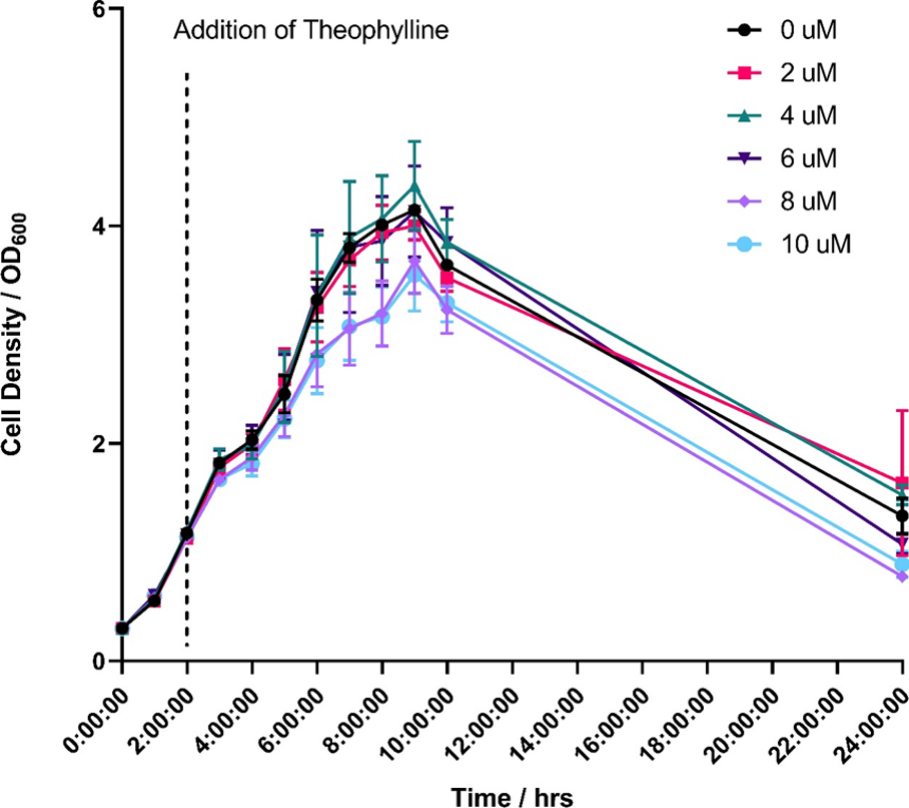
Optical density (OD_600_) of *P. thermoglucosidasius* containing the plasmid pMTL-RbxE at different concentrations of theophylline (0, 2, 4, 6, 8, and 10 mM). Statistical analysis was carried out using GraphPad Prism 9.5.1. (2-way ANOVA Tukey’s multiple comparisons test, *n* = 3). No statistical significance was observed.

After successfully confirming that the riboswitches were active in *P. thermoglucosidasius*, we proceeded to their further characterization by assessing the effect of different concentrations of theophylline on the strength of the induction and the rate of growth. First, to determine if theophylline had any effect on the growth rate, *P. thermoglucosidasius* transformed with pMTL-RbxE was grown in different concentrations of theophylline (0, 2, 4, 6, 8, and 10 mM). *P. thermoglucosidasius* was able to grow in the presence of theophylline but as the concentration increased a small reduction in growth rate was observed (Figure 4). However, this observed reduction in growth showed no statistical significance (2-way ANOVA Tukey’s multiple comparisons test, *n* = 3).

**Figure 5:**
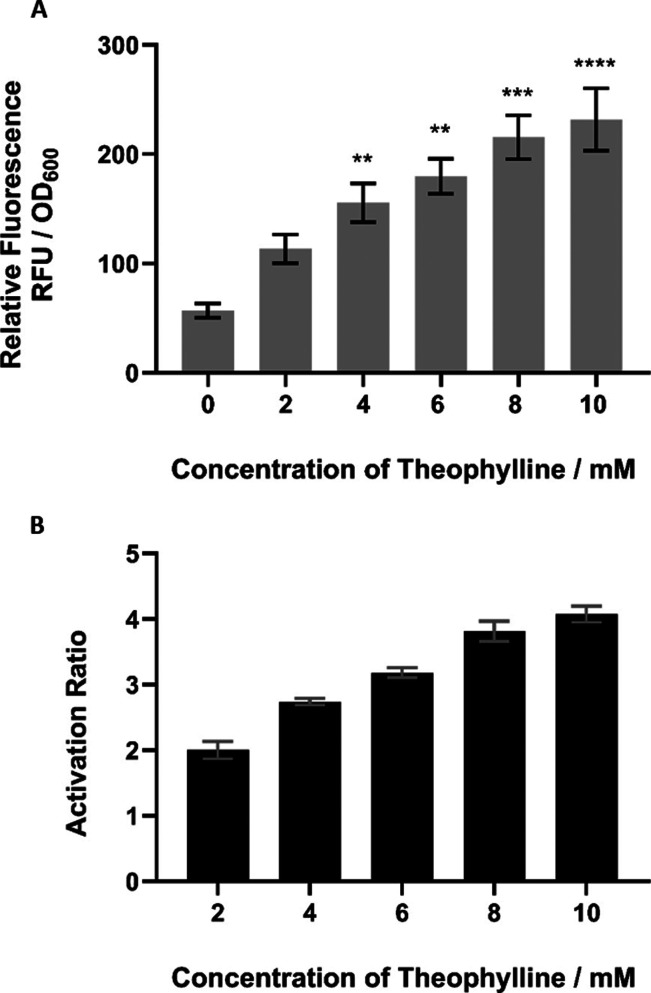
Performance of the theophylline-responsive riboswitch RbxE in *P. thermoglucosidasius* tested at increasing concentrations of theophylline (0, 2, 4, 6, 8, and 10 mM). (A) Relative strength of pMTL-RbxE at increasing concentrations of theophylline dissolved in water in *P. thermoglucosidasius* as determined by the relative fluorescence units (RFU) of sfGFP expression, normalized by cell growth (OD_600_). Error bars denote the standard error of the mean (*n* = 3). Statistical analysis was carried out using GraphPad Prism 9.5.1. *P* values ≤0.05 were considered statistically significant and are represented by asterisks. The following asterisk format is used throughout; *p ≤ 0.05, **p ≤ 0.01, ***p ≤ 0.001, ****p ≤ 0.0001 (unpaired two-tailed *t* test). (B) Activation ratio of pMTL-RbxE increased at increasing concentrations of theophylline. The activation ratio for each concentration was calculated by dividing the normalized RFU of sfGFP of the induced sample by the normalized RFU of the uninduced sample (0 mM). Error bars denote the standard error of the mean (*n* = 3).

In addition to observing the growth effects of different concentrations of theophylline, we wanted to assess the effect of increasing concentrations of theophylline on expression levels and whether the riboswitches exhibited dose dependency. To achieve this, *P. thermoglucosidasius* was transformed and cultivated with pMTL-RbxE in increasing concentrations of theophylline (0, 2, 4, 6, 8, and 10 mM) (Figure 5). We observed a clear increase in the expression of sfGFP as the concentration of theophylline increased, demonstrating a dosage-dependent response. This illustrates that gene expression can be tailored to the particular requirements of the application.

**Figure 6:**
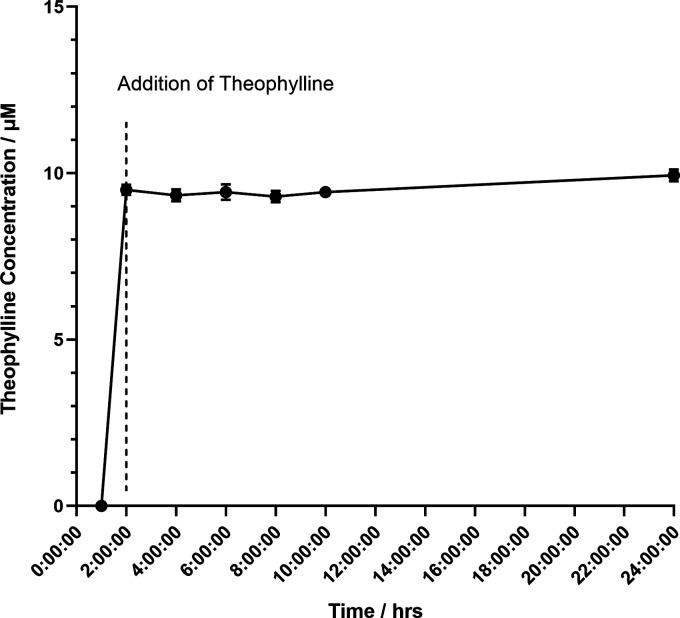
Metabolism profile of theophylline in *P. thermoglucosidasius*. Theophylline (10 mM) was added to the culture, and the concentration was monitored over a 24 h period through HPLC-UV. Error bars denote the standard error of the mean (*n* = 3).

A further requirement of the effective inducible system is that the inducer is not metabolized by the host organism. Therefore, to test this, we cultivated *P. thermoglucosidasius* in 2SPYNG medium supplemented with 10 mM theophylline. The concentration of theophylline was monitored by HPLC–UV analysis of cell-free supernatant samples over a period of 24 h. Results showed that the concentration of theophylline remained constant over the course of the experiment, indicating that theophylline is not metabolized by *P. thermoglucosidasius* (Figure 6).

### Production and Validation of RiboCas93 for Controllable, Efficient, and Directed Genome Editing

Having successfully demonstrated riboswitch-dependent controllable gene expression in *P. thermoglucosidasius*, we turned our attention to our primary aim, the development of an effective stCas93-based genome editing tool in which production of the Cas9 nuclease was controlled. To achieve this we took the previously described stCas93-based pMTL675555,^15^ in which the expression of *cas93* was constitutive and substituted the constitutive P_*ldh*_ promoter with the inducible P_*gapdh*_ RbxE riboswitch. The resultant vector was designated as pMTL-RiboCas93. To demonstrate its utility, we undertook the recreation of the industrially relevant bioethanol production strains *P. thermoglucosidasius* TM242^17^ and LS242^20^ from the progenitor, TM89 (Δ*ldhA*). First, the upregulation of pyruvate dehydrogenase (*pdhA*) was achieved through the replacement of its promoter (P_*pdhA*_) with that (P_*ldhA*_) of the lactate dehydrogenase promoter gene from *Geobacillus stearothermophilus* in the Δ*ldh* background, creating AM180 (Δ*ldhA*, *pdhA*^*up*^). Subsequently, the pyruvate formate lyase gene (*pflB*) was deleted to form the strain AM242 (Δ*ldhA*, *pdhA*^*up*^, and Δ*pflB*).

**Figure 7:**
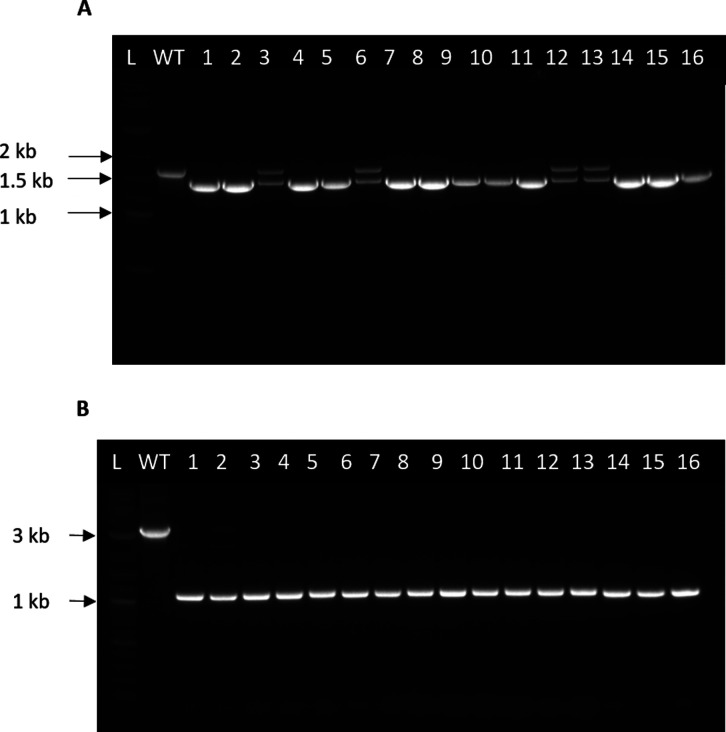
Gel electrophoresis images of colony PCRs (cPCR) to screen for RiboCas9 mutants. (A) cPCR screening of mutants for pyruvate dehydrogenase promoter (P_*pdhA*_) replacement to the lactate dehydrogenase promoter (P_*ldhA*_) from *Geobacillus stearothermophilus* using the primers Cas3_Pdh^up^_F and Cas3_Pdh^up^_R. A PCR of WT *P. thermoglucosidasius* NCIMB 11955 was performed as a control (∼1.7 kb). Colonies with successful promoter replacement would yield a band at ∼ 1.5 kb. A 2-log DNA ladder (0.1–10 kb) (NEB) was used to determine the size of each band. (B) cPCR screening of mutants for pyruvate formate lyase (*pflB*) deletion using Cas3_Pfl_F and Cas3_Pfl_R primers. A PCR of WT *P. thermoglucosidasius* NCIMB 11955 was performed as a control (∼ 3.3 kb). Colonies with successful *pflB* deletion would yield a band at ∼ 1 kb. A 2-log DNA ladder (0.1–10 kb) (NEB) was used to determine the size of each band.

The plasmid pMTL-AM180, harboring the P_*pdhA*_ to P_*ldhA*_ replacement cassette, was transformed into *P. thermoglucosidasius* TM89 (Δ*ldhA*). The transformants were selected and successful mutants were produced as reported in the Materials and Methods section, Generation of Mutants. Colony-PCR (cPCR) screening using oligonucleotide primers flanking the P_*pdhA*_ generated DNA bands of ∼1.7 and ∼1.5 kb, corresponding to the wild type (WT) and mutant alleles, respectively. To obtain pure mutants, the cells were further subjected to five sequential passages of 12 h in fresh 2SPYNG Km media with theophylline. cPCR screening of colonies demonstrated that a DNA band of a size consistent with the replacement of P_*pdhA*_, ∼1.5 kb, had occurred in 16 of 16 colonies (Figure 7a). After the subsequent passages, 4 out of the 16 colonies remained mixed colonies, with 12 of the 16 colonies producing clean replacement mutants. Sanger sequencing of the PCR products confirmed the desired replacement of P_*pdhA*_ with the P_*ldhA*_ promoter.

Having created the P_*pdhA*_ to P_*ldhA*_ replacement, the deletion of *pflB* was targeted in the AM180 (Δ*ldhA, pdh*^*up*^) strain. Thus, plasmid pMTL-AM236 containing the *pflB* deletion cassette was constructed. The protocol to achieve the P_*pdhA*_ to P_*ldhA*_ replacement was used to obtain pure *pflB* mutants, generating DNA bands of ∼ 1 kb fragment compared to ∼ 3.3 kb for the WT in 16 out of 16 colonies tested, indicating 100% editing efficiency (Figure 7b).

Here, we have successfully accomplished our primary aim, the regulation of *stCas93* to alleviate its potential metabolic burden. In addition to observing extremely high editing efficiency, RiboCas93 also demonstrated a clear advantage in terms of transformation efficiency over the original stCas93. Transformation efficiencies routinely observed when utilizing stCas93 equate to ∼1.5 × 10^1^ CFU/μg of DNA, whereas with RiboCas93 this was increased significantly to a transformation efficiency of 4.56 × 10^2^ CFU/μg of DNA. This demonstrates that we have successfully alleviated the low transformation efficiencies and/or low editing efficiencies traditionally observed when using stCas93. By developing RiboCas93 we have enabled the reliable and efficient production of stable engineered strains in *P. thermoglucosidasius* and potentially many other thermophilic bacilli.

### Characterization of the Strain AM242

**Figure 8:**
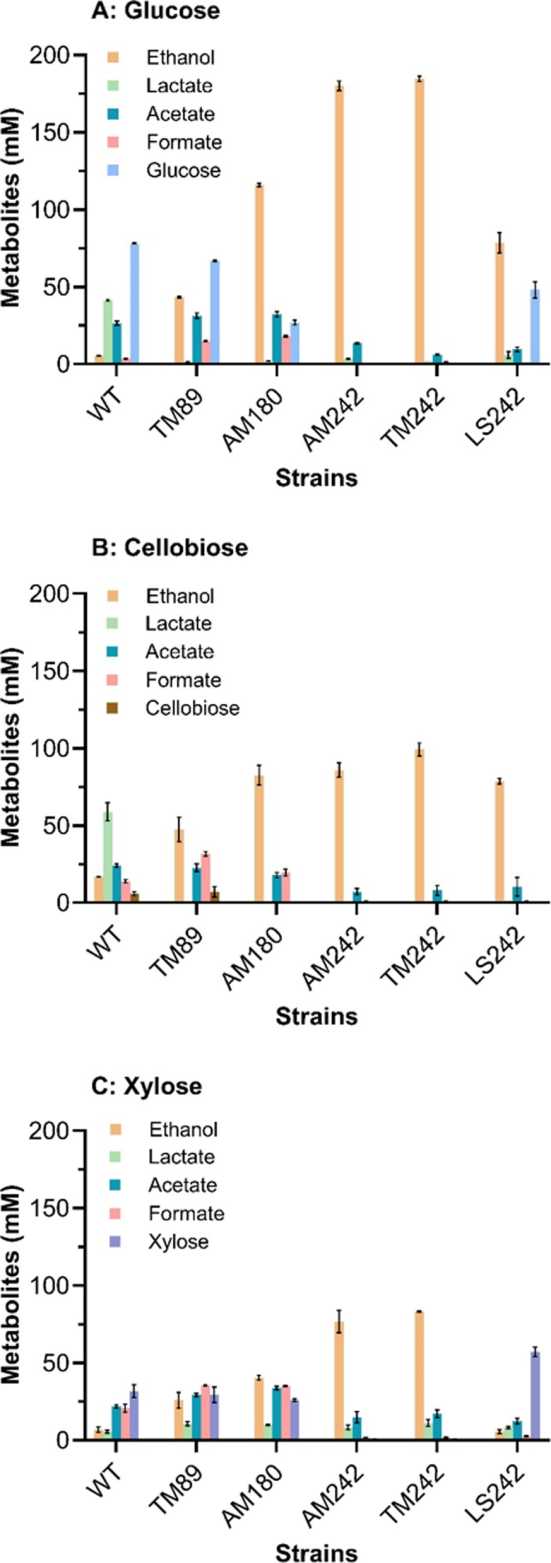
Fermentation profiles of engineered strains of *P. thermoglucosidasius* NCIMB 11955 (TM89, AM180, AM242, TM242, and LS242) showing the concentrations of the metabolites ethanol, lactate, acetate, and formate and the corresponding residual sugar concentrations (glucose, cellobiose, and xylose). WT *P. thermoglucosidasius* NCIMB 11955 was also analyzed as a control. The metabolite concentrations were determined using HPLC-UV after 24 h of growth in sealed 50 mL falcon tubes incubated at 60 °C, 250 rpm. Error bars on each graph denote the standard deviation (*n* = 3). (A) Metabolite concentration of fermentations performed using 40 mL of ASYE with 1% yeast extract and 2% glucose. (B) Metabolite concentration of fermentations performed using 40 mL of ASYE with 1% yeast extract and 1% Cellobiose (C) Metabolite concentration of fermentations performed using 40 mL of ASYE with 1% yeast extract and 1% xylose.

The fermentation profiles of the mutants created, AM180 (Δ*ldh, pdhB*^*up*^) and AM242 (Δ*ldhA, pdhA*^*up*^*,* Δ*pflB*), were evaluated alongside WT *P. thermoglucosidasius*, TM89 (Δ*ldhA*), TM24, and LS242.^17,20^ Growth of strains was performed on ASYE medium (40 mL) in Falcon tubes (see Materials and Methods for details), and the culture supernatant was analyzed using High-Performance Liquid Chromatography-Ultraviolet (HPLC-UV). The comparative fermentation profiles of the genetically equivalent strains AM242 and TM242 showed no significant differences in using glucose as a carbon source (Figure 8a) and were in agreement with those previously reported by Cripps et al. (2009). The ethanol yield of AM242 was comparable to that of TM242 (0.42 g/g glucose), representing over 80% of the theoretical yield (Table 1). One of the inherent advantages of *P. thermoglucosidasius* as a host for biofuel production such as ethanol is their ability to grow on a wide range of substrates, such as both hexose and pentose sugars typically found in lignocellulosic biomass, a feat not achieved by model organisms such as *E. coli* and *S. cerevisiae*. Thus, cellobiose and xylose were also converted to ethanol by the AM242 strain (Figure 8b,c) with yields of 0.40 g/g and 0.36 g/g, respectively, representing over 70% of theoretical yields (Table 1).

**Table 1:** Ethanol Yield of the Engineered Strains of *P. thermoglucosidasius* NCIMB 11955^a^

strains	metabolite concentration (mM) after 24 h of fermentation			
	substrate	consumed substrate	ethanol	ethanol yield (g/g)
WT (NCIMB 11955)	glucose	32.9	5.3	0.04
TM89 (Δ*ldhA*)	glucose	44.2	43.4	0.25
AM180 (Δ*ldhA*, *pdhA*^*up*^)	glucose	84.1	115.9	0.35
AM242 (Δ*ldhA*, *pdhA*^*up*^, Δ*pflB*)	glucose	111.0	180.1	0.42
TM242 (Δ*ldhA*, *pdhA*^*up*^, Δ*pflB*)	glucose	111.0	184.6	0.43
LS242 (Δ*ldhA*, *pdhA*^*up*^, Δ*pflB*)	glucose	63.0	78.6	0.32
AM242 (Δ*ldhA*, *pdhA*^*up*^, Δ*pflB*)	cellobiose	29.2	85.9	0.40
TM242 (Δ*ldhA*, *pdhA*^*up*^, Δ*pflB*)	cellobiose	29.2	99.3	0.46
LS242 (Δ*ldhA*, *pdhA*^*up*^, Δ*pflB*)	cellobiose	29.2	78.7	0.36
AM242 (Δ*ldhA*, *pdhA*^*up*^, Δ*pflB*)	xylose	66.6	77.6	0.36
TM242 (Δ*ldhA*, *pdhA*^*up*^, Δ*pflB*)	xylose	66.6	83.5	0.39
LS242 (Δ*ldhA*, *pdhA*^*up*^, Δ*pflB*)	xylose	12.8	4.4	0.10

a Initial glucose: 111.0 mM; initial cellobiose: 29.2 mM; initial xylose: 66.6 mM.

When generating a mutant, often one problem that requires attention is the production of single nucleotide polymorphisms (SNPs). Often, these changes in the DNA are caused when the production of the mutant requires multiple rounds of screening and passages to both produce the mutant and subsequently lose the editing plasmid. These can cause issues, especially when trying to produce a desired product. This was highly evident in a study published by Sheng et al. (2017)^20^ when producing the ethanol-producing strain LS242. Here, they observed a significant difference in ethanol production between strains TM242 and LS242. On further inspection, the authors observed clear differences in SNPs between the two strains, noting that 11 SNPs were produced in TM242 and 4 in LS242.^17,20^

To counter these issues, the method developed here using RiboCas93 has significant advantages over the methods previously employed to produce mutants. First, the use of an inducible promoter allows for the toxic selection pressure, which may lead to SNPs, to be alleviated during transformation and early growth phases. Second, the method involved in producing mutants involves the growth of mutants in liquid culture instead of restreaking a single colony, which is more liable to fix random SNPs in the population. To determine our hypothesized advantages, we sequenced the AM242 strain. The sequencing data revealed that no additional SNPs were produced when creating AM242 using RiboCas93, demonstrating a major advancement over previous methods of strain production.

Here we have demonstrated the implementation of an inducible CRISPR/Cas9 system based on theophylline-responsive riboswitch E^28,29,35^ to facilitate efficient gene knockout (KO) and knock-in (KI) in *P. thermoglucosidasius*. This was achieved by the generation of an engineered ethanol production strain (AM242) equivalent to those previously published^17,20^ without the production of additional mutations (SNPs or Indels). This involved upregulation of the *pdhA* gene by replacing its native promoter with P_*ldhA*_ from *G. stearothermophilus* in a Δ*ldhA* strain (TM89) and deletion of the *pflB* gene. The genetically equivalent strain (AM242) produced high titers of ethanol comparable to TM242.^17^

To the best of our knowledge, this study is the first implementation of a theophylline-responsive CRISPR/Cas9 genome editing system in *P. thermoglucosidasius* for genome engineering. Thus, the method represents a considerable improvement and adds to the existing tools available for the metabolic engineering of *P. thermoglucosidasius* and thermophilic bacilli generally to produce fuels and value-added chemicals.

## Materials and Methods

### Biological Materials and Growth Conditions

The bacterial strains used in this study are listed in Table S1.

*E. coli* Top10 strain (Invitrogen) was used as a cloning host and grown in LB media (5 mL) or on LB agar plates at 37 °C and supplemented with kanamycin (50 μg/mL). LB broth containing tryptone (10 g), yeast extract (5 g), and NaCl (5 g) per liter of deionized water. The pH of the medium was adjusted to 7.5 using HCl or NaOH prior to autoclaving. LB agar was prepared by the addition of No. 1 bacteriological agar (10 g) per liter of deionized water before being autoclaved.

For general growth, *P. thermoglucosidasius* NCIMB 11955 (TMO Renewables) was grown in 2SPYNG (10 mL) at 52 °C shaking (250 rpm) or on TSA agar plates at 52 °C, O/N. Where appropriate, kanamycin (12.5 μg/mL) was used for the selection of plasmids. 2SPYNG contained soy peptone (16 g), yeast extract (10 g), and NaCl (5 g) per liter of deionized water. The pH was adjusted to 7.0 using 5 M KOH prior to autoclaving. TSA agar was prepared using TSA (40 g) (Sigma-Aldrich) per liter of deionized water and autoclaved.

The growth of bacterial cultures in a liquid medium was monitored by measuring optical density at 600 nm (OD_600_) using a Jenway 6300 (Cole-Parmer). Samples measured were diluted to 1 × 10^–1^.

For the transformation of *P. thermoglucosidasius*, 2SPY was used. This was produced in the same way as 2SPYNG but included glycerol (10 g). The pH was adjusted to 7.0 using 5 M KOH prior to autoclaving.

For the characterization of the ethanol-producing strains, *P. thermoglucosidasius* was grown in ASYE (40 mL) at 60 °C in Falcon tubes. ASYE was prepared according to Cripps et al. (2009) and NaH_2_PO_4_·2H_2_O (10 mM), K_2_SO_4_ (10 mM), citric acid (2 mM), MgSO_4_·7H_2_O (1.25 mM), CaCl_2_·2H_2_O (0.02 mM), Na_2_MoO_4_·2H_2_O (1.65 mM), (NH_4_)2SO_4_ (20 mM), ZnSO_4_·7H_2_O (25 mM), FeSO_4_·7H_2_O (100 mM), MnSO_4_·H_2_O (50 mM), CuSO_4_·5H_2_O (5 mM), CoSO_4_·7H_2_O (10 mM), NiSO_4_·6H_2_O (16.85 mM), H_3_BO_3_ (6.5 mM), biotin (12 mM) and 1% yeast extract, per liter of deionized water. Filter-sterilized bis-tris (40 mM), PIPES (40 mM), and HEPES (40 mM) were added after autoclaving. The appropriate sugar was added as indicated, and the pH was adjusted to 7.0.

For curing of editing plasmids, the growth condition of *P. thermoglucosidasius* NCIMB 11955 were changed. The temperature was raised from 52 to 60 °C and the selection pressure of kanamycin was removed. The cultures were grown in 2SPYNG (10 mL) at 60 °C in a Falcon tube for 12 h before being passaged into fresh media six successive times. The cultures were then serially diluted to 1 × 10^–4^ and plated onto TSA plates and grown O/N to produce single colonies. These single colonies were then screened to identify the plasmid deficient strain by replica plating onto TSA plates with and without kanamycin.

### Reagents

All PCR reactions were performed using a Phusion 2X Master Mix (New England Biolabs) or DreamTaq Green PCR Master Mix (Thermo Fisher Scientific). T4 ligase (Promega) was used for the DNA ligation reactions. Restriction enzymes were purchased from Thermo Fisher Scientific.

### Plasmid Design and Construction

Oligonucleotide primers were synthesized by Sigma-Aldrich and are listed in Table S2. Plasmids were constructed by restriction enzyme-based cloning procedures.^43^ Constructs were verified by DNA sequencing (Source Bioscience UK Limited). All of the plasmids used in this study are listed in Table S3 and may be sourced from www.plasmidvectors.com. The plasmid pMTL61110^20^ was used as the base chassis for all of the plasmids constructed in this study. Homology cassettes used in this study were produced using splicing by an overlap extension polymerase chain reaction (SOE-PCR).

### Oligonucleotide Design, Analysis, and Synthesis

Oligonucleotides for synthetic guide RNA (sgRNA), polymerase chain reaction (PCR), and sequencing of DNA sequences were designed manually. The secondary structure of oligonucleotides was analyzed for the production of primers and for calculating the free energy of Rho-independent terminators. DNA sequences were analyzed using the OligoAnalyzer tool (https://www.idtdna.com/calc/analyzer) of Integrated DNA Technologies. Oligonucleotide synthesis was performed by Sigma-Aldrich. Spacer acquisition for sgRNA was completed using Benchling CRISPR Guide Design software (www.benchling.com).

### Transformation of Plasmid DNA

All *E. coli* stains were transformed using a heat-shock method. Plasmid DNA (1–2 μL) (100–500 ng/μL) was added to chemical competent *E. coli*. Top 10 cells (60 μL). This mixture was left on ice for 30–45 min, then transferred to 42 °C for 30 s, then returned back to ice for 3–5 min. LB media (900 μL) was then added to the cells and left to recover for 1.5 h, incubated at 37 °C, and shaking (200 rpm). After recovery, the cells (200 μL) were plated onto LB agar plates containing the appropriate antibiotic selection.

Transformation of *P. thermoglucosidasius* was conducted following the procedure described by Cripps et al. (2009).^17^ Plasmid DNA (5 μL) (100–500 ng/μL) was added to electrocompetent *P. thermoglucosidasius* NCIMB 11955 cells (60 μL) in a precooled 1 mm gap electroporation cuvette. Electroporation was carried out using a GenePulser (BioRad), set at 600 Ω, 2.5 kV, and 10 μFD. Immediately following the pulse, prewarmed 2SPY (1 mL) was added to the cells and left to recover for 2.5–4 h, incubated at 52 °C shaking (250 rpm). After recovery, the cells were concentrated by centrifugation (5000*g*, 5 min) and resuspended in 2SPY media (200 μL) before being plated onto prewarmed TSA agar plates containing kanamycin (12.5 μg/mL).

### Riboswitch Characterization

For characterization of the theophylline-dependent riboswitches, sGFP was used to determine the relative levels of gene expression. *P. thermoglucosidasius* was grown in 2SPYNG Km (50 mL) at 52 °C in a baffled conical flask for 8 h. Theophylline was diluted in either DMSO (48 mg/mL) or water (25 mg/mL). Theophylline was added to the cultures at the early exponential phase (OD_600_ = ∼1.0) and diluted to the appropriate concentration. After 8 h of growth, samples (1 mL) were taken and the cells were harvested via centrifugation (15, 000×*g*, 1 min) and the supernatant was discarded. The cells were then washed once using phosphate-buffered saline (PBS) (l mL), the cells were again harvested and the supernatant was discarded before being resuspended in PBS (700 μL). Three technical replicates (200 μL) of each biological replicate were used and plated into a clear bottom black 96-well plate. The level of sfGFP was measured using a microplate reader (CLARIOstar) using the excitation and emission values.^15,44^ The relative fluorescence intensities (RFU) were then normalized using the optical density (OD_600_) of each sample.

### Generation of Mutants

For the generation of mutants using theophylline-dependent CRISPR/Cas9 technology, the required plasmid was first transformed into *P. thermoglucosidasius*. The transformants were then selected and incubated (52 °C) on TSA plates supplemented with kanamycin (Km) (12.5 μg/mL). The subsequent colonies were individually picked and cultured (52 °C) in 2SPYNG (10 mL) with Km and theophylline (8 mM) overnight (O/N) to induce stCas93 production. These cultures were subsequently passaged and grown in 2SPYNG Km (10 mL) at 52 °C in a falcon tube for 12 h until a clean mutant was produced. The cultures were then diluted and plated on TSA Km plates to obtain discrete colonies. Plasmid loss was carried out using 2SPYNG (10 mL) without antibiotic selection at 60 °C.

### Analytical Methods

Theophylline metabolism profile in *P. thermoglucosidasius* was analyzed using high-performance liquid chromatography (HPLC-UV). Sample preparation involved adding 1-volume ice-cold methanol to supernatant samples and aqueous calibration standards and stored overnight (−20 °C) with pulsed vortex mixing. Solid cleared by centrifugation (5 min 15,000×*g*). Samples were diluted 4-fold with H_2_O and analyzed using HPLC with UV absorbance detection. Samples (10 μL) were chromatographed (Thermo Scientific; Ultimate 3000) in reversed-phase mode (Waters CORTECS 2.7 μm T3, 2.1 mm × 50 mm) at 0.25 mL min^–1^, using a binary gradient of methanol in 10 mM ammonium acetate (water) with UV (280 nm) absorbance detection. Instrument control and data processing used Chromeleon software (Thermo Scientific; 7.2.10).

Metabolites produced by *P. thermoglucosidasius* were analyzed using HPLC-UV, performed using the Dionex UltiMate 3000 System. 5 mM H_2_SO_4_ was used as the mobile phase. The sample supernatant was mixed, in a 1:1 ratio, with diluent, which consisted of the mobile phase mixed with 50 mM valeric acid. Samples were filtered to remove particulate matter through 0.2 μm syringe filters directly into HPLC vials containing 300 μL inserts with split caps. The samples were run on the Bio-Rad Aminex HPX-87H 300 mm × 7.8 mm × 9 μm column, at a flow rate of 0.5 mL min^–1^, at 35 °C for 55 min.

## Supporting Information Available

Bacterial strains used in this study; primers used in this study; plasmids used in this study; and construction of pMTL-AM180 and pMTL-AM236 plasmids (PDF)

## Abbreviations

SNPs: single nucleotide polymorphisms
CRISPR: clustered regularly interspaced short palindromic repeats
Cas: CRISPR-associated protein
stCas93: *Streptococcus thermophilus* cas93
Indels: insertions/deletions
RBS: ribosomal binding sequence
sfGFP: superfolder green fluorescent protein
DMSO: dimethyl sulfoxide
RFU: relative fluorescence units
*ldhA*: lactate dehydrogenase
*pdhA*: pyruvate dehydrogenase
P_*padhA*_: *pdhA* promoter
P_*ldhA*_: *ldhA* promoter
*pdh*^*up*^: *pdh* upregulated
*pflB*: pyruvate formate lyase
Km: kanamycin
cPCR: colony polymerase chain reaction
O/N: overnight
WT: wild type
HPLC-UV: high-performance liquid chromatography-ultraviolet
KO: knock-out
KI: knock-in
SOE-PCR: splicing by overlap extension polymerase chain reaction
sgRNA: synthetic guide RNA
PCR: polymerase chain reaction
PBS: phosphate buffered saline
