# Supplementary material for: Parageobacillus thermoglucosidasius Strain Engineering Using a Theophylline Responsive RiboCas for Controlled Gene Expression
Source: ACS Synth Biol. 2024 Mar 22;13(4):1237–45. doi: 10.1021/acssynbio.3c00735 (PMC11036489; doi:10.1021/acssynbio.3c00735)
Supplement: Supplementary file 1 — sb3c00735_si_001.pdf [file sb3c00735_si_001.pdf]

Parageobacillus thermoglucosidasius strain engineering using a theophylline responsive RiboCas for controlled gene expression

Matthew S. H. Lau[1#], Abubakar Madika[1,2#], Ying Zhang[1], Nigel P. Minton[1,3*]

[1]BBSRC/EPSRC Synthetic Biology Research Centre (SBRC), Biodiscovery Institute, School of Life Sciences, University of Nottingham, University Park, Nottingham, NG7 2RD, United Kingdom

[2]Department of Microbiology, Faculty of Life Sciences, Ahmadu Bello University, Zaria 810107, Nigeria

[3]NIHR Nottingham Biomedical Research Centre, Nottingham University Hospitals NHS Trust and The University of Nottingham, Nottingham, United Kingdom

*Corresponding Author Email: Nigel.minton@nottingham.ac.uk

SUPPLEMENTARY INFORMATION

Table S1. Bacterial strains used in this study.

Strain	Description	Reference
Parageobacillus thermoglucosidasius NCIMB11955	Wild Type Isolate	TMO Renewables, London, UK
Escherichia coli Top10	F- *mcr*A Δ(*mrr-hsdRMS-mcrBC*) Φ80*lacZM15*Δ*lacX74 deoR recA1araD139* Δ(*ara-leu)*7697*galU galK rpsL* (Str[R])*endAl nupG*	Invitrogen Ltd
P. thermoglucosidasius TM89	Δ*ldhA*	Cripps et al.[1]
P. thermoglucosidasius AM180	Δ*ldhA*, *pdhA[up]*	This study
P. thermoglucosidasius AM242	Δ*ldhA*, *pdhA[up]*, Δ*pflB*	This study
P. thermoglucosidasius TM242	Δ*ldhA*, *pdhA[up]*, Δ*pflB*	Cripps et al.[1]
P. thermoglucosidasius LS242	Δ*ldhA*, *pdhA[up]*, Δ*pflB*	Sheng et al.[2]

Table S2. Primers used in this study. Uppercase denotes annealing base pairs. Lowercase denotes non-annealing base pairs. Red denotes restriction endonuclease sites.

Name of Primer	Sequence (5' – 3')	Description
**Riboswitch Cloning**		
Gapdh_F_NotI	gatcgcggccgcGCTTTTTTTATGATTTTACTGAATTTTTCTTCACATATCA	Amplification of P_{gapdh}
Gapdh_R_BsaI	cccggtctctattgATGCTATACTAATTAACAGCATTTCACAAAAATAGTATATC	
Rbx_F_BsaI	ggtctcaCAATACGACTCACTATAGGTGATACCAGC	Amplification of Riboswitch
RbxE_R_BsaI	ccggtctccctcatCTTGTTGTTACCTCCTTAGCAGGG	
RbxF_R_BsaI	ccggtctccctcatTAAGTTACCTCCTTAGCAGGGTGC	
RbxG_R_BsaI	ccggtctccctcatTAACACACCTCCTTAGCAGGGTG	
RbxK_R_BsaI	ccggtctccctcatCCTCCTTAGCAGGGTGCTGC	
sfGFP_F_BsaI	tataggtctcaATGAGCAAAGGCGAGGAACTG	Amplification of sfGFP
sfGFP_R_XhoI	atatctcgagttaGCTACCCTTATACAATTCGTCCATACCATG	
**RiboCas93 Cloning**		
XbaI_Pgapdh_F	gatctctagaGCTTTTTTTATGATTTTACTGAATTTTTTCTTCACATATCACC	Amplification of P_{gapdh} RbxE
RbxE_BsaI_R	ccggtctcTCTTGTTGTTACCTCCTTAGCAGGG	
Cas93_BsaI_F	ccggtctcACAAGATGACTAAGCCATACTCAATTGGACTTG	Amplification of stCas93
Cas93_R	gtcaCCTGCAGGTTAACCCTCTCCTAG	
**Screening Primers**		
Cas3_Pfl_F	CAGTTTCCGGTGTTTTTTCTCATCG	Amplification of *pflB* gene region for screening of deletion
Cas3_Pfl_R	GTTCCGCAAACGCTTAAGCCC	
Cas3_Pdhup_F	GCAGCGGTTTATCTGGTTGAC	Amplification of *pdhA* promoter region for screening of promoter replacement
Cas3_Pdhup_R	GATGCCAGGAATTCCCGCT	

Table S3. Plasmids used in this study.

Plasmid	Description	Reference
pMTL61110	*P. thermoglucosidasius* modular shuttle vector version 1, ColE1 + tra, pUB110, km^R	Sheng et al.[2]
pMTL_RbxX	pMTL61110 containing P_{gapd}, the specific Riboswitch (E, F, G or K), sGFP gene	This study
pMTL_sfGFP	pMTL61110 containing T1T2 terminator, P_{gapd}, synthetic RBS, sfGFP gene	Lau et al.[3]
pMTL_TRsG	pMTL61110 containing T1T2 terminator, promoter-less, synthetic RBS, sfGFP gene	Lau et al.[3]
pMTL675555	pMTL61110 containing *stcas9-3* under the control of P_{ldh} and the *ldh* RBS, P_{gapd}, T1T2 terminator	Lau et al.[3]
pMTL-RiboCas93	pMTL675555, containing *stcas9-3* under the control of the inducible P_{gapdh} RbxE riboswitch	This study
pUC57-Kan-Cas3-Pdhup	Vector harboring *pdhA* promoter upregulation cassette	Azenta Life Science, Ltd
pUC57-Kan-Cas3-Pfl	Vector harboring *pflB* gene deletion cassette	Azenta Life Science, Ltd
pMTL-AM180	pMTL-RiboCas93 containing P_{pdhA} targeting sgRNA under the control of P_{gapdh}, T1T2 terminator downstream of the sgRNA and the *G. stearothermophilus* 165 bp *ldhA* promoter homology cassette	This study
pMTL-AM236	pMTL-RiboCas93 containing *pflB* targeting sgRNA under the control of P_{gapdh}, T1T2 terminator downstream of the sgRNA and a *pflB* gene editing homology cassette	This Study

Construction of pMTL-AM180 and pMTL-AM236 plasmids

To produce the vectors pMTL-AM180 and pMTL-AM236, the application-specific modules for both *pdhA* upregulation and *pflB* deletion were synthesised and located between BamHI and AscI restriction sites of a pUC57-Kan vector by Azenta Life Sciences Ltd (formerly GENEWIZ, Ltd), yielding plasmids pUC57-Kan-Cas3-pdhup and pUC57-Kan-Cas3-pfl, respectively.

Up-regulation of *pdhA* involved replacing its native 337 bp promoter fragment with the *G. stearothermophilus* 165 bp *ldhA* promoter, P_{ldh}. Therefore, for *pdhA* upregulation, the module contained sgRNA targeting P_{pdhA} (identified using Benchling CRISPR Guide Design software (www.benchling.com), T1T2 terminator, P_{ldh} from *G. stearothermophilus* flanked by an editing template comprising homology arms (450 bps) from up and downstream of the P_{pdhA}.

The module for *pflB* deletion contained sgRNA targeting *pflB* gene (identified using Benchling CRISPR Guide Design software (www.benchling.com), T1T2 terminator, and an editing template comprising fused left and right homology arms (450 bps), corresponding to the regions up and downstream of the *pflB* gene.

Using restriction enzyme-based cloning procedures the fragments were cloned into pMTL-RiboCas93 to produce the completed vector, pMTL-AM180 and pMTL-AM236 respectively.

REFERENCES

[1] Cripps, R. E., Eley, K., Leak, D. J., Rudd, B., Taylor, M., Todd, M., Boakes, S., Martin, S., and Atkinson, T. (2009) Metabolic engineering of *Geobacillus thermoglucosidasius* for high yield ethanol production, *Metab Eng 11*, 398-408.

[2] Sheng, L., Kovács, K., Winzer, K., Zhang, Y., and Minton, N. P. (2017) Development and implementation of rapid metabolic engineering tools for chemical and fuel production in *Geobacillus thermoglucosidasius* NCIMB 11955, *Biotechnol Biofuels 10*, 5.

[3] Lau, M. S. H., Sheng, L., Zhang, Y., and Minton, N. P. (2021) Development of a Suite of Tools for Genome Editing in Parageobacillus thermoglucosidasius and Their Use to Identify the Potential of a Native Plasmid in the Generation of Stable Engineered Strains, *ACS Synth Biol 10*, 1739-1749.
